# Aging-Related Immune Cell Phenotypes and Mortality in the Framingham Heart Study

**DOI:** 10.21203/rs.3.rs-3773986/v1

**Published:** 2023-12-26

**Authors:** Ahmed A.Y. Ragab, Margaret F. Doyle, Jiachen Chen, Yuan Fang, Kathryn L. Lunetta, Joanne M. Murabito

**Affiliations:** Boston University School of Public Health; University of Vermont, Larner College of Medicine; Boston University School of Public Health; Binghamton University, State University of New York; Boston University School of Public Health; Boston University Chobanian & Avedisian School of Medicine

**Keywords:** Immune cell phenotype, Aging, Immunosenescence, Inflammaging, T cells, Immune system, All-cause mortality, Cardiovascular mortality, Non-cardiovascular mortality

## Abstract

**Background:**

The global increase in human life expectancy is evident. The total number of individuals aged 60 or above is anticipated to reach 2 billion by 2050. Aging, an inherently complex process, manifests prominently in the changes observed in the immune system. A notable marker of immune system aging is the presence of Aging-Related Immune Cell Phenotypes (ARIPs). Despite their significance, the connections between various ARIPs and mortality have not been thoroughly investigated. We prospectively investigated 16 different ARIPs using flow cytometry, namely, CD4/CD8 ratio, Granzyme B + CD8/Granyzme B + CD4, T_N_/T_M_ = Tn / (Teff + Tem + Tcm) for T_N_/T_M_ CD4 + and T_N_/T_M_ CD8 + ratios, Th17/CD4 + Treg, Tc17/CD8 + Treg, Th17, Tc17, CD4 + Temra, CD8 + Temra, CD4 + CD25 + FoxP3+ (CD4 + Treg), CD8 + CD25 + FoxP3+ (CD8 + Treg) CD4 + CD27−, CD4 + CD28-CD27−, CD8 + CD27−, CD8 + CD28-CD27− and IL-6 in relation to survival outcome among dementia-free Framingham Heart Study (FHS) offspring cohort participants who attended the seventh exam (1998–2001).

**Results:**

Among 996 participants (mean age 62 years, range 40 to 88 years, 52% female), the survival rate was 65% during 19 years of follow-up. For the model adjusting for age, sex, and cytomegalovirus (CMV) serostatus, higher CD4/CD8 and Tc17/CD8 + Treg ratios were significantly associated with lower all-cause mortality (HR:0.86 [0.76–0.96], 0.84 [0.74–0.94], respectively) and higher CD8 regulatory cell levels (CD8 + CD25 + FoxP3+) were associated with higher all-cause mortality (HR = 1.17, [1.03–1.32]). Higher IL-6 levels were associated with higher all-cause, cardiovascular, and non-cardiovascular mortality (HR = 1.43 [1.26–1.62], 1.70 [1.31–2.21], and 1.36 [1.18–1.57], respectively).

## Background

Advancements in medical science and public health initiatives have ameliorated the health and well-being of populations, resulting in an increase in life expectancy. It’s anticipated that over 2 billion individuals will be 60 years or older by 2050.[1] The changing demographics of the world are presenting significant challenges to healthcare systems globally, for instance the global healthcare expenditure is expected to reach 15 trillion dollars by the midpoint of the current century.[2] This underscores the importance of better understanding of the inherently complex process of aging in order to tackle the challenges coming with these demographic fluctuations.

Chronic, subtle inflammation known as “inflammaging” is one of the twelve key elements of aging.[3] Alongside other aging hallmarks such as immunosenescence, they are considered major drivers for many age-related chronic health conditions such as diabetes, neurodegenerative diseases, and cancer.[4,5] There is compelling evidence that inflammaging and immunosenescence induce each other, resulting in a state of mutual maintenance. Age-related changes have been observed in both the innate and adaptive immune systems.[6,7] In the realm of the aging adaptive immune system, the T cell compartment experiences the most profound changes.[8] An increased accumulation of T memory and T effector cells along with a decline in T cell repertoire and number of naïve cells are the defining characteristics of those changes.

A variety of cell types and cell ratios have been used to help identify the shift in the immune system with age, and they have collectively been called age-related immune phenotypes (ARIP). One such measure, the CD4/CD8 ratio, is affected not only by age, but also by other factors such as sex and CMV serostatus. [9,10] A recent report has shown that specific CD4 + T cell and CD8 + T cell subsets can be a valid alternative to CD4/CD8 ratio as an ARIP measure.[11] The ratio of CD4 + naïve T-cells to the sum of CD4 + T_CM_ (Central Memory), T_EM_ (Effector Memory), and T_EFF_ (Effector) cells [CD4T_N_/T_M_] showed a strong association with biological age and 2-year mortality, while CD8T_N_/T_M_ showed an association with chronological age but not mortality.

In our recent study we profiled a broad panel of 116 immune cell phenotypes and observed associations of various T cell subsets with age, sex and CMV serostatus.[12] Both CD4T_N_/T_M_ and CD8T_N_/T_M_ were significantly associated with age, yet little is known about their role in mortality. Th17 cells promote immune responses through proinflammatory cytokine secretion, while CD4 + Tregs act as immune regulators by suppressing various immune cells, maintaining immune tolerance, and preventing excessive inflammation. The balance between these two subsets is crucial for immune system function. The imbalance between Th17 and CD4 + Treg cells, which are rival and interlinked factors, plays a significant role in organ-specific immunity and predicted short-term mortality in certain disorders.[13–15] While neither Th17 nor CD4 + Tregs are associated with age in our data,[12] little is known about the impact of this imbalance on mortality in a community-based cohort.

Various reports have examined the CD4/CD8 ratio with survival outcomes, generally among older adults. In an older British cohort (n ~ 500), a CD4/CD8 ratio < 1.0 showed worse survival outcomes compared to CD4/CD8 ratio > 1 with an age adjusted hazard ratio of 1.47, but lost significance after adjusting for both age and sex.[16] An elevated CD4/CD8 ratio above five in the BELFRAIL study (n = 235, mean age 86.7 years) was found to be associated with higher all-cause mortality among octogenarian Belgian women who tested negative for CMV, although survival bias cannot be ruled out.[17] In a Spanish cohort (n = 328, aged 85 years old), an inverted CD4/CD8 ratio (CD4/CD8 < 1) showed no increased risk for mortality.[18] The relationship between ARIPs other than the CD4/CD8 ratio and risk of death are not yet well established. Moreover, most studies of the CD4/CD8 ratio have had relatively small sample sizes, with a short time of follow up and without cause specific survival analysis.

IL-6 has gained recognition as a pivotal mediator in the immune response, particularly due to its direct impact on CD4 and CD8 T cells and its role in shaping their functions.[19,20] Elevated IL-6 levels are associated with cardiovascular events and mortality in community cohorts (including the Framingham Heart Study) and COVID-19 hospitalized individuals.[21–25].

We selected 16 ARIPs from our panel of immune cell phenotypes as well as relative plasma IL-6 levels to investigate their association with all-cause mortality, cardiovascular mortality, and non-cardiovascular mortality in a sample of Framingham Heart Study Offspring (FHS) participants aged 40 years and older with long-term follow-up.

## Results

### Study participants characteristics

The study included 996 participants with an average age of 62 years (range: 40 to 88) at the time of immune cell profiling, and 48% were males (n = 480). As indicated in Table 1, 14% were current smokers (n = 139), 14% had prevalent cardiovascular disease (n = 142), 34% had hypertension (HTN, n = 343) and 6% had diabetes (n = 61).

The median time of follow up was 19 years [IQR:15–20]. During the follow up, 353 participants (35%) died. A total of 81 participants (8%) died due to cardiovascular causes, and 272 participants (27%) died due to non-cardiovascular causes. Descriptive statistics for the ARIPs and IL-6 are in the Supplemental Table 1.

### CMV and mortality

A total of 465 (47%) participants were CMV positive, with an average age of 63 years (± 9, 46% males). During their follow up, 193 CMV-positive participants (42%) died. We investigated the relationship between CMV status and mortality adjusting for age, sex, and level of education. CMV status did not show a significant effect on all-cause mortality, cardiovascular mortality, or non-cardiovascular mortality (Table 2; all p > 0.05). Additionally, we investigated the impact of CMV status on mortality among participants over and under the age of 60 years, but there was no significant effect observed in either age group. Separate analyses stratifying the participants based on sex and CMV status did not affect the risk of mortality in either sex (Table 2).

### Survival analysis (Model 1)

**Figure 1:**
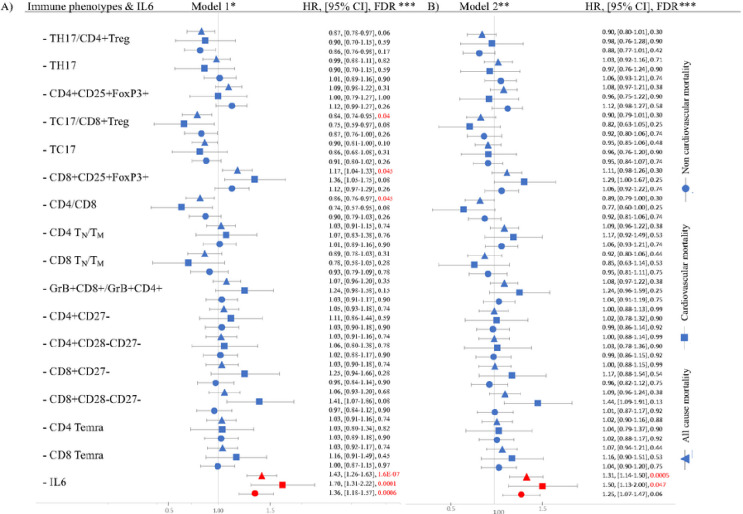
Forest plot demonstrating the associations between aging related immune cell phenotypes (ARIP) or IL-6 and mortality: Model 1 (panel A) and Model 2 (panel B). Hazard ratio (HR) is reported per 1 standard deviation increase in the phenotype. • * Model is adjusted for age, sex and CMV status. • ** Model is adjusted for age, sex, CMV status, prevalent cardiovascular disease, prevalent cancer, diabetes, HTN, use of lipid lowering medications, body mass index (BMI) and smoking status. • *** Data represented as HR, 95% confidence interval and FDR. We set FDR<0.05 to be significant.

We employed Cox models to investigate individually the association of each of the 16 different immune cell phenotypes and IL-6 with mortality, while accounting for age, sex, and CMV status (Model 1, Fig. 1A and Supplemental table 2). Higher Tc17/CD8 + Treg ratio had a protective effect against all-cause mortality (HR = 0.84, FDR = 0.04), partially driven by a deleterious effect of higher CD8 regulatory cells (CD8 + CD25 + FoxP3+, HR = 1.17, FDR = 0.045). Higher CD4/CD8 ratio was protective against all-cause mortality (HR = 0.86, FDR = 0.045). Finally, higher IL-6 levels were associated with higher all-cause, cardiovascular, and non-cardiovascular mortality (HR = 1.43, 1.70, and 1.36 (all FDR < 0.01), respectively). In the participants over 60 years group, higher IL-6 levels showed association with higher all-cause, cardiovascular mortality and non-cardiovascular mortality after adjusting for model 1 covariates (Supplemental table 7). After adding prevalent cancer to model 1 covariates, the level of significance remained (Supplemental table 8). However, after adding cardiovascular risk factor to model 1, the effect got attenuated for cardiovascular mortality (Supplemental table 9). In the CMV positive group, after adjusting for age and sex, higher IL-6 showed association with higher all-cause and cardiovascular mortality (Supplemental table 11). The same results were shown after adjusting for age, sex, and prevalent cancer (Supplemental table 12). However, the effects got attenuated after adjusting for age, sex, and cardiovascular risk factors (Supplemental table 13).

### Survival analysis (Model 2)

Association effects were attenuated, and immune cell phenotype associations were no longer significant after adding prevalent cancer and the cardiovascular risk factor covariates: prevalent cardiovascular disease, diabetes, hypertension (HTN), use of lipid lowering medications, body mass index (BMI) and smoking status (Fig. 1B and Supplemental Table 5). While also modestly attenuated, the association between IL-6 and all-cause mortality and cardiovascular mortality remained significant after adjustment for all covariates with HRs of 1.3 (FDR = 0.0005) and 1.5 (FDR = 0.047) respectively.

To further explore the attenuation of effects, we found that adding only prevalent cancer as a covariate into Model 1 did not attenuate the immune cell associations (Supplemental table 3.), while adding the set of cardiovascular risk factors to Model 1 did (Supplemental table 4).

**Figure 2:**
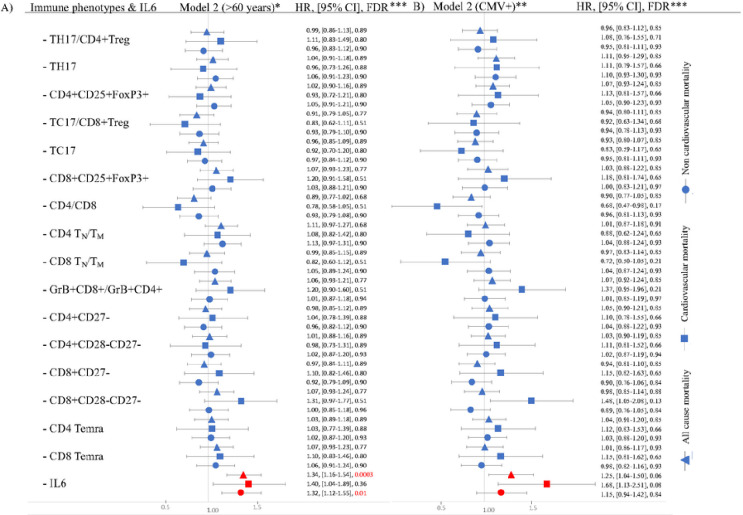
Forest plot demonstrating the associations between aging related immune cell phenotype (ARIP) or IL-6 and mortality for participants over 60 years of age (Panel A) and positive CMV status (Panel B) using Model 2 covariates. Hazard ratio (HR) is reported per 1 standard deviation increase in the phenotype. • * Model is adjusted for age, sex, CMV status, prevalent cardiovascular disease, prevalent cancer, diabetes, HTN, use of lipid lowering medications, body mass index (BMI) and smoking status • ** Model is adjusted for age, sex, prevalent cardiovascular disease, prevalent cancer, diabetes, HTN, use of lipid lowering medications, body mass index (BMI) and smoking status. • *** Data represented as HR, 95% confidence interval and FDR. We set FDR<0.05 to be significant.

Analysis of participants over 60 years of age at the time of the immune cell and IL-6 measurements (Fig. 2A and Supplemental table 6) or participants who were CMV positive (Fig. 2B and Supplemental table 10), yielded no significant associations for the ARIPs. In the CMV positive group (Fig. 2B), CD8 + CD28-CD27− phenotype had a trend for increased risk for cardiovascular mortality with HR of 1.48 (95% CI: 1.05– 2.08, FDR:0.13), while higher CD4/CD8 ratio had a trend for lower risk for cardiovascular mortality with HR of 0.68 (95% CI: 0.47– 0.98, FDR = 0.17). Higher IL-6 was associated with higher risk for all-cause and non-cardiovascular mortality in the age > 60 subset (Fig. 2A) and had a trend for higher all-cause and cardiovascular mortality in the CMV + subset (Fig. 2B).

## Discussion

The main findings from this study of ARIPs and mortality in the Framingham Offspring study are that CMV seropositivity is not associated with all-cause mortality, cardiovascular mortality or non-cardiovascular mortality, even when stratified by age and sex. Higher Tc17/CD8 + Treg and CD4/CD8 ratios had a protective effect against all-cause mortality, and higher IL-6 levels showed an association with more all-cause, cardiovascular, and non-cardiovascular mortality. Additionally, while many of the ARIPs and IL-6 were associated with mortality in the minimally adjusted model, after adjustment for CVD risk factors and comorbidities only IL-6 was significantly associated with mortality. These observations suggest that a cardiovascular pathway explains at least some of the association between ARIPs and mortality.

In our study, we saw no association of CMV with mortality after adjusting for age, sex, and educational level. Although many reports described an association between all-cause mortality and CMV serostatus, recent large multicenter cohort studies showed no such association.[26–28] Most studies that reported a positive association of CMV with mortality had relatively older participants (80 + years of age) or used a minimally adjusted model for only age and sex.[29,30]

Our age, sex, and CMV status adjusted analyses revealed that higher Tc17/CD8 + Treg ratio and CD4/CD8 ratio were associated with a lower risk of all-cause mortality. Phillips et al also observed protective effects of higher CD4/CD8 ratio on all-cause mortality in 4256 middle-aged men (HR = 0.58, 95% CI 0.41–0.81). [31] While little data exists on the role of the Tc17/CD8 + Treg ratio in aging and mortality, it is thought to be an important marker of the balance between immune protection and pathology. As illustrated in Mills review on IL17 and IL17 producing cells, Tc17 cells serve a protective role against intracellular bacteria, fungi, and small parasites by producing IL17.[32] If this process is left unchecked, inflammatory pathways remain activated. The presence of regulatory cells that produce immunosuppressive cytokines (i.e., IL10, TGF-beta, IL35) suppress the effector cells (i.e. Tc17) to decrease inflammation. And while the exact role of the CD8 + Tregs as we have defined them (CD8 + CD25 + FoxP3+) may not be the most prevalent type of CD8 + regulatory cell, the presence of CD25 on the surface makes these cells more likely to respond to the pro-inflammatory IL2, which is believed to cause an immunosuppressive response by enhancing Fas-mediated activation-induced cell death. [33,34]

Higher levels of CD8 + CD25 + FoxP3+ (CD8 + Tregs) cells and IL-6 showed higher risk for all-cause mortality, with IL-6 also associated with cardiovascular and non-cardiovascular mortality. In the fully adjusted model, which adjusts for prevalent disease and known risk factors, only IL-6 levels were associated with mortality. Since most previous studies were done in older populations, we further examined only participants over 60 years of age and only elevated IL-6 remained a risk factor for all-cause and non-cardiovascular mortality. In the CMV positive group, higher CD8 + CD28-CD27− cells and lower CD4/CD8 ratio showed a non-significant trend for cardiovascular mortality. The presence of CD28 and CD27 on CD8 cells has been implicated in primary response to viruses and their loss may be indicative of repeated viral challenges over a lifetime.[35,36]

Known as the “gerontologist’s cytokine” IL-6 has been considered as a major aging related biomarker. There is strong evidence that IL-6 is associated with aging and chronic diseases.[37,38] Moreover, IL-6 was associated with 10-year mortality and cardiovascular events in several cohorts.[24,39] We report a strong association between IL-6 and mortality with long term follow up (HR = 1.50 in fully adjusted model, similar to data previously reported in this cohort (HR = 1.41).[25] There are two main differences in the current study. First, IL-6 measurements in this study utilized the OLINK Target 96 inflammation panel which reports data as a relative concentration, compared to previous data that used a quantitative ELISA, but the results were similar despite different follow up times, indicating the utility of the OLINK IL-6 proteomic data.[25] Second, our fully adjusted model includes CMV, which was not included in the previous study. The higher hazard ratio observed in the CMV positive participants (HR = 1.68) may indicate a role for CMV in the IL-6 association with mortality.

The strength of our report relies on several factors. The FHS Offspring cohort is a well characterized community-based study with long term continued surveillance, broad availability of clinical data, and the availability of death records which allowed for a cause specific mortality sub-analysis for cardiovascular and non-cardiovascular mortality. Limitations for our study include the lack of racial and ethnic diversity, since FHS Offspring participants are predominantly White of European ancestry, the lack of longitudinal data for the laboratory-based measures, and the use of cryopreserved cells rather than fresh cells, although increasing pools of evidence suggest their usefulness in this type of study.[40,41]

## Conclusion

Examining a cohort of 996 participants over a span of 19 years, our report explored the association between ARIPs, IL-6 levels, and mortality. Notably, higher Tc17/CD8 + Treg and CD4/CD8 ratios were linked to lower all-cause mortality. Moreover, the CD8 + CD25 + FoxP3+ (CD8 + Treg) phenotype showed heightened risks for all-cause mortality. Most strikingly, elevated IL-6 levels consistently demonstrated robust positive associations with amplified risks of mortality particularly in the CMV positive participants. Collectively, these findings provide a nuanced perspective on the intricate relationships underlying immune phenotype and mortality dynamics, suggesting a potential for future research and clinical implications.

## Methods

### Study Sample

The Framingham Heart Study (FHS) is a well-characterized prospective cohort study. It commenced in 1948 with the recruitment of 5209 individuals, forming the Original cohort.[42] Subsequently, in 1971, the FHS expanded its reach by enrolling the Offspring cohort, which comprised 5129 participants. This cohort consists of the offspring of the Original cohort participants as well as their spouses.[43] Since their enrollment, the Offspring cohort participants have undergone regular examinations every 4–8 years. Notably, during the seventh examination conducted between 1998 and 2001, a total number of 3539 Offspring participants were present.

We selected 1332 participants from the Offspring cohort who attended examination 7 and had at least 2 vials of stored peripheral blood mononuclear cells (PBMCs) available so that the resource would not be exhausted. From these, we identified a study sample of 1000 dementia free individuals aged 40 years and older. Of these, we excluded participants with samples that failed quality control for having too much missing data, and participants missing cytomegalovirus serostatus. The ethical guidelines outlined in the 1964 Declaration of Helsinki were followed in conducting this study. The participants in the study provided written consent before each examination. The exams conducted as part of the FHS were reviewed and granted approval by the Institutional Review Board at Boston University Medical Center.

### Immunophenotyping Methods

Immune cell phenotyping protocols have been previously described.[12] Briefly, cryopreserved PBMCs from the Framingham Heart Study Offspring Cohort Exam 7 were thawed, diluted, washed, and resuspended in phosphate-buffered saline. Cells were filtered and divided into 5 assay panels for immunophenotyping. For surface labeling panels cells were incubated with fixable live/dead stain followed by fluorescent antibodies. Cells were pelleted, washed, fixed with paraformaldehyde, and stored in the dark at 4°C.

For intracellular staining, cells were stimulated with phorbol myristate acetate/Ionomycin in the presence of Brefeldin A. After washing, cells were incubated with CD3/CD4/CD8 antibodies, fixed with paraformaldehyde, and incubated with antibodies or isotype controls in the presence of saponin. The cells were washed and stored in 2% paraformaldehyde at 4°C until flow cytometry analysis. For regulatory phenotypes, cells were incubated with CD3/CD4/CD8 antibodies, fixed with paraformaldehyde, then intracellularly stained, as previously described.[12]

Flow cytometry was performed on a MACSQuant 16 flow cytometer with machine compensation set using single color compensation beads. Isotype controls and fluorescence-minus-one (FMO) controls aided gate setting. FCS Express 6.0 software was used for data analysis. Vericell PBMC control sample assessed run variability with coefficient of variation: CD3 + cells (2.5%), CD4 + cells (12.3%), CD8 + cells (17.5%). Primary data was reported as a percent of their main parent population (ie CD4 + for CD4 subtypes) and gating strategies were previously illustrated.[12]

We selected 16 immune cell phenotypes for investigation supported by a review of relevant literature. [13,44–47] Six immune cell phenotypes ratios have been calculated, namely, CD4/CD8 ratio, Granzyme B + CD8/Granyzme B + CD4, Tn/Tm = Tn / (Teff + Tem + Tcm) for Tn/Tm CD4 + and Tn/Tm CD8 + ratios, Th17/CD4 + Treg, and Tc17/CD8 + Treg.[11] Descriptive statistics for these immune cells are in the Supplemental table 1, and complete descriptions of the immune cells are described elsewhere.[12]

### Cytomegalovirus

A plasma sample collected at Offspring Exam 7 and stored at −80C existed for 943 of the 1000 Offspring exam 7 participants on which we also profiled the immune cells. We performed CMV IgG assay by ELISA (Creative Diagnostics CMV IgG kit Catalog # DEIA326R). Quantitative values in U/ml were obtained, and the CV for the CMV assay was determined to be 5.9%. CMV status was categorized into: CMV negative/equivocal (≤ 15 U/ml) and CMV positive (> 15 U/ml). Participants with missing CMV measures have been excluded from statistical analysis.

### Interleukin-6

We measured IL-6 using the OLINK inflammation panel on plasma samples. The protein expression levels, in Normalized Protein eXpression (NPX) units, measured a relative quantification unit on *log*_2_ scale, with one NPX difference indicating a doubling of protein concentration. The OLINK NPX Signature software was used for quality control and normalization of data. Additional details concerning quality control of this data in the FHS Offspring is described elsewhere.[48] Descriptive statistics for IL-6 in this sample are in the Supplemental table.

### End point events

The outcome events in this study were all cause mortality and cause specific mortality which was categorized into cardiovascular mortality and non-cardiovascular mortality. Cardiovascular mortality was defined as death due to coronary heart disease, stroke, or other cardiovascular causes; individuals who died of non-cardiovascular causes were censored at time of death. Non-cardiovascular mortality was defined as death from causes other than cardiovascular diseases such as cancer, other, or unknown causes, and cardiovascular deaths were censored at time of death. In order to determine the cause of death, multiple sources of information were reviewed by a panel of FHS investigators such as medical records, personal physician notes, nursing home records, death certificates, interviews with surviving family members, and if available autopsy data. The death events were dated and coded from exam 7 (1998–2001) until the end of 2019.

### Covariates

All covariates were recorded during the exam 7 visit (1998–2001). For cardiovascular diseases, all records were adjudicated by a panel of three senior physicians using standard criteria and all available evidence. [49] For cerebrovascular diseases, a review committee analyzed comprehensive records including assessments by a neurologist from FHS.[50] Prevalent cardiovascular disease was defined as coronary heart disease (including myocardial infarction, angina pectoris, and coronary insufficiency), cerebrovascular disease (including stroke and transient ischemic attack), intermittent claudication, and congestive heart failure. Diabetes was defined by meeting one of the following criteria: having a fasting blood glucose level equal to or exceeding 126 mg/dL, having a random blood glucose level equal to, or exceeding 200 mg/dL, or utilizing antidiabetic medications. Hypertension was defined as systolic BP ≥ 140 or a diastolic BP ≥ 90 or being under anti-hypertensive treatment. Smoking status was defined by self-report of smoking one or more cigarettes per day in the year preceding exam 7. Body mass index was calculated by dividing weight in kilograms by the square of height in meters. Prevalent cancer occurred prior to exam 7 and majority of self-reports were confirmed by a thorough examination of pathology reports, with two independent investigators reviewing the associated medical records. Using lipid-lowering medications was also included as a covariate.

### Statistical Analysis

For descriptive analysis, the baseline characteristics of the sample have been summarized in the form of mean and standard deviation or mean and range (continuous and normally distributed variables), median and interquartile range (continuous and not normally distributed variables) and proportions (categorical variables). We used Cox proportional hazards models to estimate hazard ratios (HRs) and corresponding 95% confidence intervals (CIs) for the effect of immune cells phenotypes on hazard of death, adjusting for covariates (described below). The end point of the study was set to December 31, 2019. The follow-up period for each participant was calculated from the time of their examination (1998–2001) until death or being right censored at the end of 2019 and reported in years.

For each immune cell phenotype, we performed a rank-based inverse normal transformation by converting the ranks of the phenotype value into quantiles from a standard normal distribution. We accounted for family relationships using a robust variance clustered on family ID. Our primary model adjusted for age at baseline (exam 7), sex and CMV status (Model 1). A secondary model (Model 2) also adjusted for age, sex, CMV status, and included prevalent cardiovascular disease, prevalent cancer, diabetes, HTN, use of lipid lowering medications, BMI and smoking status. In the sensitivity analyses, prevalent cancer, and the set of cardiovascular risk covariates were considered separately with the Model 1 covariates.

We repeated analyses in the subgroup of participants age > 60 at exam 7 and separately in the subgroup of participants who were CMV positive (> 15 U/ml). We used a significance threshold of false discovery rate (FDR) less than 0.05 to determine statistical significance within each model and for the subgroup analyses. All analyses were conducted in R software version 4.0.2.

## Figures and Tables

**Table 1:** Sample Characteristics

Demographic	Study sample (n = 996)
Age, mean (range)	62 (40, 88)
Sex; Female, n (%)	516 (52%)
CMV positive, n (%)	465 (46.7%)
BMI, kg/m2, (sd)	28 (5)
Attended college, n (%)	733 (73.6%)
Smoking, n (%)	139 (14%)
HTN, n (%)	343 (34.4%)
Diabetes, n (%)	61 (6.1%)
Prevalent CVD, n (%)	142 (14.3%)
Lipid lowering medications, n (%)	225 (22.6%)
SBP mmHg, mean (sd)	127 (18)
DBP mmHg, mean (sd)	74 (10)
Total cholesterol mg/dL, mean (sd)	199 (37)
Prevalent cancer, n (%)	94 (9%)

BMI: body mass index; CMV: cytomegalovirus; HTN: hypertension; CVD: cardiovascular disease; SBP: systolic blood pressure; DBP: diastolic blood pressure.

**Table 2:** CMV status and mortality^1^

All sample (n = 996)^2^	HR	Lower 0.95	Upper 0.95
all-cause mortality	0.98	0.78	1.25
cardiovascular mortality	0.66	0.41	1.08
non-cardiovascular mortality	1.11	0.85	1.45
Over 60 year old (n = 539)^3^			
all-cause mortality	1.00	0.77	1.30
cardiovascular mortality	0.71	0.41	1.20
non-cardiovascular mortality	1.11	0.82	1.51
Under 60 year old (n = 457)			
all-cause mortality	1.06	0.64	1.74
cardiovascular mortality	0.50	0.14	1.86
non-cardiovascular mortality	1.24	0.72	2.13
Male (n = 480)^4^			
all-cause mortality	1.16	0.85	1.58
cardiovascular mortality	0.74	0.38	1.43
non-cardiovascular mortality	1.31	0.92	1.87
Female (n = 516)			
all-cause mortality	0.77	0.54	1.11
cardiovascular mortality	0.53	0.26	1.10
non-cardiovascular mortality	0.87	0.58	1.31

1. Table values are HR and 95% CI levels.

2. Model adjusted for age, sex and education level.

3. Sample stratified (age≥60) and adjusted for covariates.

4. Sample stratified (sex) and adjusted for covariates.

## Data Availability

The data from this study can be requested from the External Data Repository of the Framingham Heart Study (https://www.framinghamheartstudy.org/fhs-for-researchers/data-available-overview/).

## Abbreviations

ARIPs: Aging-Related Immune Cell Phenotypes
FHS: Framingham Heart Study
CMV: Cytomegalovirus
CD4 + T_CM_: Central Memory
CD4 + TEM: Effector Memory
CD4 + TEFF: Effector
HTN: Hypertension
BMI: Body mass index
PBMCs: Peripheral blood mononuclear cells
FMO: Fluorescence-minus-one
NPX: Normalized Protein eXpression
HRs: Hazard ratios
CIs: Confidence intervals
FDR: False discovery rate
